# Synthesis and Studies of PAM-Ag-g/WS_2_/Ti_3_C_2_T_x_ Hydrogel and Its Possible Applications

**DOI:** 10.3390/polym17192588

**Published:** 2025-09-24

**Authors:** Anar Arinova, Danil W. Boukhvalov, Arman Umirzakov, Ekaterina Bondar, Aigul Shongalova, Laura Mustafa, Ainagul Kemelbekova, Elena Dmitriyeva

**Affiliations:** 1 Laboratory of Photovoltaic Phenomena and Devices, Institute of Physics and Technology, Satbayev University, Almaty 050013, Kazakhstan; a.umirzakov@sci.kz (A.U.); bondar@sci.kz (E.B.); a.shongalova@sci.kz (A.S.); kemelbekova@sci.kz (A.K.); dmitriyeva@sci.kz (E.D.); 2 Department of Chemistry, Nanjing Forestry University, Nanjing 210037, China; danil@njfu.edu.cn; 3 Department of Materials, National Center of Space Research and Technology JSC, Almaty 050010, Kazakhstan; l.mustafa@spaceres.kz

**Keywords:** dichalcogenides, MXene, WS_2_, hydrogel, flexible sensor, strain sensor, DFT, interfaces

## Abstract

In this study, a new hybrid hydrogel based on PAM (polyacrylamide)-Ag-g/WS_2_/Ti_3_C_2_T_x_ was synthesized by radical polymerization using a conductive heterostructural nanocomposite WS_2_/Ti_3_C_2_T_x_. The synergy between the polymer matrix and the interface between two-dimensional nanomaterials ensured the production of a hydrogel with high extensibility and conductivity, as well as sensory characteristics. The composite hydrogel exhibited excellent strain-sensing capabilities, with gauge factors of 1.4 at low strain and 2.8 at higher strain levels. In addition, the material showed a fast response time of 2.17 s and a short recovery time of 0.46 s under cyclic stretching, which confirms its high reliability and reproducibility. The integration of Ti_3_C_2_T_x_ and WS_2_ promoted the formation of a conductive network in the hydrogel structure, which simultaneously increased its mechanical strength and signal stability under variable loads. Measurements confirm some potential of the PAM-Ag-g/WS_2_/Ti_3_C_2_T_x_ composite hydrogel as a flexible wearable strain sensor. Based on measured numbers, we discussed the impact of the WS_2_/Ti_3_C_2_T_x_ interface on the Gauge factor and conductivity of the composite. Theoretical modeling demonstrates significant changes in the electronic structure of the WS_2_/Ti_3_C_2_T_x_ interface, and especially the WS_2_ surface, induced by substrate strain. Possible applications of the peculiar properties of PAM-Ag-g/WS_2_/Ti_3_C_2_T_x_ composite were proposed.

## 1. Introduction

Transition metal dichalcogenides (TMDs), which have excellent mechanical and electrical conductivity, are among the most promising 2D materials for the creation of a generation of smart and flexible electronic devices [1]. Among TMDs, tungsten disulfide WS_2_ is a notable candidate due to its large number of active sites and good electrical properties, and is also less toxic than graphene oxide and can therefore be used as a safer alternative in future applications [2]. The properties of TMDs can be improved by chemical modification. Various methods, such as cocatalyst loading, doping, and heterostructure design, have been used to improve the electrical conductivity of TMDs and expand their application [3,4,5]. WS_2_ TMDs possess out-of-plane sulfur atoms that readily interact with surrounding chemical groups, making it easy to form heterostructures with other 2D or 1D nanomaterials, determining the growth and morphology of heterostructured nanocomposites [6]. MXene, a 2D multilayer material, exhibits excellent conductivity and can serve as a good conductive substrate material for the uniform growth of 2D dichalcogenide sheets on top of and between layers. The development of MXene-based hybrid systems is in its infancy compared to other established 2D materials. MXene-based hybrids with interesting hierarchical structures and excellent performances have attracted considerable interest [7,8].

Conductive nanomaterial-filled hydrogels offer excellent conductivity, elasticity, ease of fabrication, and durability, making them ideal for flexible sensors [6]. Driven by Internet of Things (IoT) advancements, the market for flexible electronics like wearable sensors has expanded significantly. Conductive hydrogel-based sensors translate human mechanical motion (pressure, strain, axial displacement) into electrical signals for real-time monitoring [7]. These sensors, combined with AI, enable data analysis and predictive capabilities. However, the limited mechanical strength of traditional conductive hydrogels has hindered their broader use in flexible electronics. For instance, Jia Pan et al. [1] successfully integrated MoS_2_ into a flexible hydrogel-based sensor with a quick response time of 150 ms. Integrating WS_2_/Ti_3_C_2_T_x_ heterostructure into a hydrogel matrix is a promising approach for developing next-generation flexible wearable strain sensors. Mxene has exceptional metallic conductivity, hydrophilicity, and tunable surface end groups (–O, –OH, –F), which facilitate strong interfacial interaction with other nanomaterials and hydrogel networks. The resulting WS_2_/Ti_3_C_2_T_x_ heterointerface creates a 2D conductive network with an electric field, improving charge carrier mobility and sensor sensitivity. Recent reports demonstrate that interfacing WS_2_ with Ti_3_C_2_T_x_ can yield 2D-2D heterostructures with improved charge transport and interfacial contact and synergistic sensing/electrocatalytic behaviors [9]. However, most WS_2_/Ti_3_C_2_T_x_ studies focus on dry films/electrodes embedding a pre-formed WS_2_/Ti_3_C_2_T_x_ heterostructures nanofiller directly into hydrogel networks remains comparatively underexplored.

PAM-Ag-g hydrogel, a composite of synthetic and natural polymers, is promising for flexible sensors, biomedical devices [10], and wearable technology [11]. Agar-agar (Ag-g) provides structural integrity and prevents dehydration by forming a physical gel network that retains significant water [12]. This combination creates a dual network structure exhibiting high stretchability, elasticity, structural stability, and water retention capacity [13]. In this work, we aimed to synthesize and characterize a PAM-Ag-g/WS_2_/Ti_3_C_2_T_x_ hydrogel strain sensor with enhanced mechanical strength and sensitivity through interfacial engineering of a WS_2_/Ti_3_C_2_T_x_ heterostructure. 

## 2. Materials and Methods

### 2.1. Materials

Titanium aluminum carbide 312 MAX Phase (Ti_3_AlC_2_, ≥90%, ≤40 μm particle size, Sigma-Aldrich, Merck KGaA, Darmstadt, Germany), tangsten hexachloride (WCl_2_, ≥99% Sigma-Aldrich, MilliporeSigma, Urbana, IL, USA), L-cysteine (C_3_H_7_NO_2_S, Sigma-Aldrich, Buchs, Switzerland), ethanol (C_2_H_5_OH, Sigma-Aldrich, Taufkirchen, Germany), phosphoric acid (≥89%, Sigma-Aldrich, Buchs, Switzerland), Acrylamide (AM, CH_2_=CHCONH_2_, 99%, Sigma-Aldrich, Buchs, Switzerland), N,N′-methylene bisacrylamide (MBA, 99%, Sigma-Aldrich, Wuxi, China), Ammonium persulphate (APS, 98.5%, Sigma-Aldrich, Milwaukee, WI, USA), Agar (Ag-g, (C_12_H_18_O_9_)_n_ Sigma-Aldrich, Buchs, Switzerland).

### 2.2. Materials Characterization

The chemical structure of the samples was analyzed using Fourier transform infrared spectroscopy (FTIR, Nicolet iS10 FT-IR Spectrometer, Thermo Fisher Scientific Inc., Ogden, UT, USA). The surface chemical composition of MXene/WS_2_ was investigated via X-ray Photoelectron Spectroscopy (XPS, NEXSA Thermo Scientific). Scanning electron microscopy (SEM-EDS, JSM-7500F, JEOL Ltd., Yamagata, Japan) was used to study the morphology of sample surfaces. The crystal structures of samples were analyzed by XRD (SmartLab, Rigaku Co., Takatsuki, Japan, Cu Kα radiation, λ = 0.154056 nm). XRD data were obtained in the 2θ range from 20 to 80 °C at a scan rate of 6 deg. min^−1^ using X-ray radiation 40 kV, 30 mA. Raman spectroscopy (The Horiba LabRam Evolution, Palaiseau, France) was used to study the structure and phase composition of the hybrid WS_2_/Ti_3_C_2_T_x_.

### 2.3. Synthesis

Synthesis of Ti_3_C_2_T_x_ MXene. Ti_3_C_2_T_x_ was prepared by selectively etching 1.0 g Ti_3_AlC_2_ powder was slowly added to 20 mL 48 wt% HF solution in a Teflon beaker under a fume hood with appropriate protective equipment. The mixture was stirred for 48 h at room temperature. The resulting mixture was then washed with deionized (DI) water until a neutral pH was reached. Finally, the Ti_3_C_2_ MXene was dried at 60 °C overnight in a vacuum oven.

Synthesis of WS_2_. During the synthesis, 0.099 g (0.01 M) of WCl_6_ and 0.061 g (0.02 M) L-cysteine were dissolved in 25 mL of distilled water under magnetic stirring at 400 rpm for 30 min at room temperature. The above solution was transferred to a 50 mL autoclave chamber. Then, the teflon-lined stainless-steel autoclave was heated at 180 °C for 24 h in a vacuum oven. The product was collected by centrifugation and repeated washing with ethanol and distilled water. The sample was dried at 60 °C overnight in a hot air oven. The powder was collected by centrifugation and repeated washing with ethanol and distilled water. The sample was dried at 600 °C overnight in a hot air oven.

**Figure 1:**
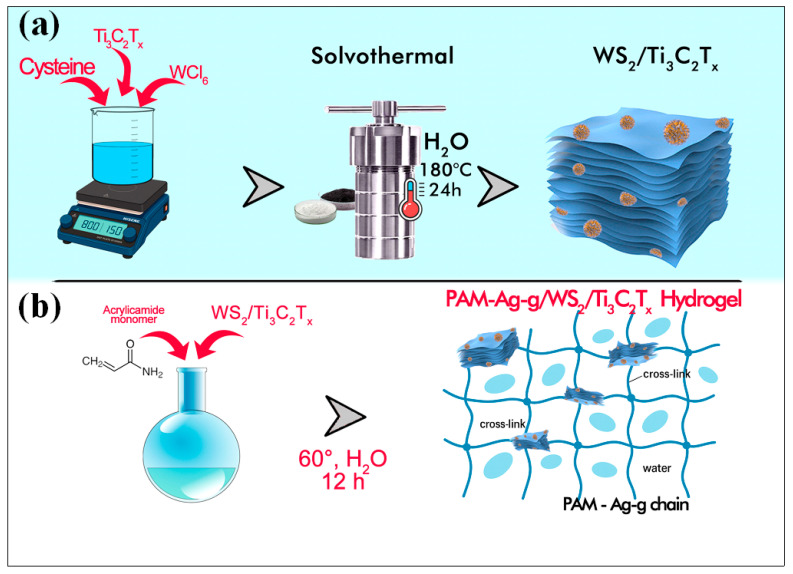
Schematic illustration of the synthesis process of (**a**) WS_2_/Ti_3_C_2_T_x_ and (**b**) PAM-Ag-g/WS_2_/Ti_3_C_2_T_x_ Hydrogel.

Synthesis of WS_2_/Ti_3_C_2_T_x_. The layered hybrid structure WS_2_/Ti_3_C_2_ was synthesized via a one-step hydrothermal method. A solution containing 0.005 M WCl_6_, 0.02 M cysteine, and Ti_3_C_2_ was added to distilled water and stirred for 30 min at room temperature. The resulting mixture was then heated at 180 °C for 24 h in a 50 mL Teflon-lined autoclave. The black precipitate was collected by centrifugation, washed three times with distilled water, and dried in a vacuum oven at 60 °C for 12 h. A schematic illustration is presented in Figure 1a. 

Synthesis PAM-Ag-g/WS_2_/Ti_3_C_2_T_x_ Hydrogel. PAM-Ag-g/WS_2_/Ti_3_C_2_T_x_ hydrogel was synthesized via free radical polymerization. Acrylamide (1.5 g) and agar (400 mg) were dissolved in 10 mL of deionized water, followed by the addition of N,N′-methylenebisacrylamide (0.03 g) as a crosslinker and ammonium persulfate (0.03 g) as an initiator. After homogenization, WS_2_/Ti_3_C_2_T_x_ nanofillers (0.2–1 wt% relative to total polymer solids), pre-dispersed in 2 mL of deionized water by bath sonication for 30 min was introduced. The mixture was then polymerized in a mold at 60 °C for 12 h to form the hydrogel (Figure 1b). A schematic illustration is presented in Figure 1b. 

### 2.4. Mechanical Testing of PAM-Ag-hg/WS_2_/Ti_3_C_2_T_x_ Hydrogel

The mechanical properties of the sample were evaluated using a tensile testing machine (Materials Testing Machine Z010/TN2S, Kennesaw, GA, USA), and the tests were performed at a tensile rate of 10 mm/min. For tensile testing, the hydrogels were made in the form of a rectangle (45 mm long, 20 mm high) and stretched at a strain rate of 5 mm min^−1^. The strength was calculated by integrating the area under the stress–strain curve.

### 2.5. Sensor Characteristic of PAM-Ag-g/WS_2_/Ti_3_C_2_T_x_ Hydrogel

The sensor characteristics of the hydrogel were evaluated using a custom-made platform with a linear motor (LinMot PS01-37x120F-HP-C, Dongguan, China) operating at 5–11 Hz in contact-opening mode to simulate compression and decompression. Each end of the hydrogel was inserted into a copper wire connected to a TH LCR meter, and the sensors were adjusted to the LCR meter (LCR-106X, IVYTECH, Changzhou, China) at different resistor deformations. The output resistance was measured using Kitstar software (version 5.2, Kitware Inc., Clifton Park, NY, USA) at different strains, and a force sensor (Vernier LabQuest Mini, Beaverton, OR, USA) monitored the applied strain.

### 2.6. First-Principles Modeling

The atomic structure and energetics of various configurations were studied using DFT with the QUANTUM-ESPRESSO code [14] and the GGA-PBE [15] functional, taking into account van der Waals forces corrections [16]. For all calculations, we used ultrasoft pseudopotentials [17]. The values of energy cutoffs of 35 Ry and 400 Ry for the plane-wave expansion of the wave functions and the charge density, respectively.

## 3. Results and Discussion

### 3.1. Chemical Characteristics of WS_2_/Ti_3_C_2_T_x_

**Figure 2:**
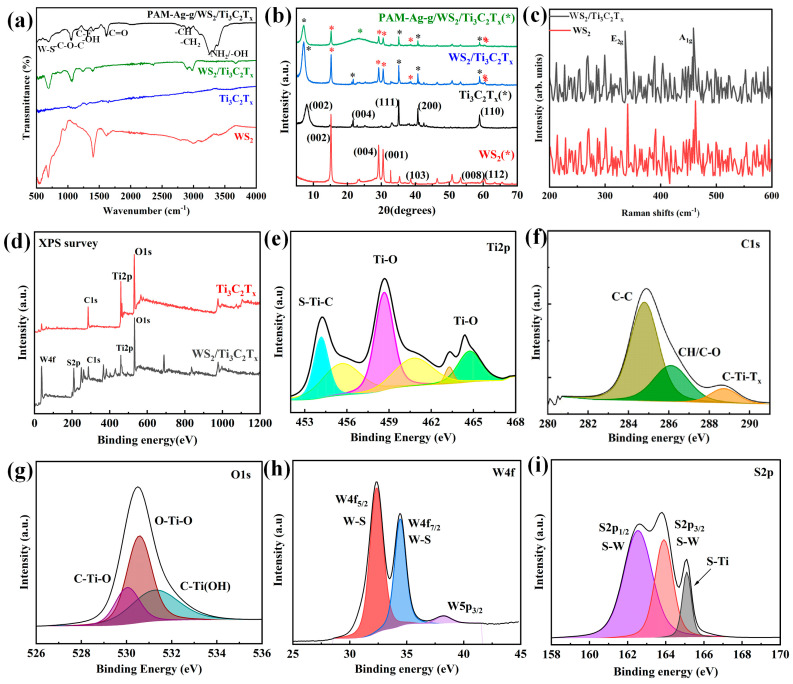
(**a**) FTIR spectra of pure WS_2_, Ti_3_C_2_T_x_, and hybrid structure WS_2_/Ti_3_C_2_T_x_; (**b**) XRD patterns of WS_2_, Mxene and, hybrid structure WS_2_/Ti_3_C_2_T_x_; (**c**) Raman spectra of WS_2_ and WS_2_/Ti_3_C_2_T_x_; (**d**) XPS survey spectrum; (**e**) Ti 2p; (**f**) XPS spectra of C 1s; (**g**) XPS spectra of O 1s; (**h**) XPS Spectra of W 4f; (**i**) XPS Spectra of S 2p.

The FTIR spectroscopy analysis (Figure 2a) allowed us to identify the functional groups in the synthesized PAM-Ag-g/WS_2_/Ti_3_C_2_T_x_ hydrogel composite, and to compare its spectral characteristics with those of pristine Ti_3_C_2_T_x_, WS_2_ and WS_2_/Ti_3_C_2_T_x_ nanocomposite. The composite spectrum exhibits enhancement and broadening in the higher-wavenumber region 3200–3500 cm^−1^, indicating the –NH_2_ group of PAM [18], –OH group of Ti_3_C_2_T_x_ [19], and –OH group of agar-agar. The asymmetric stretching vibration of –CH_2_ for PAM is evident at ~3050 cm^−1^ [20]. A noticeable shift in this band, as well as shifts in other characteristics peaks, can be attributed to the strong interfacial interactions and hydrogen bonding between PAM chains and the WS_2_/Ti_3_C_2_T_x_ [18]. The peaks at 2990 cm^−1^ and 2890 cm^−1^ were apparently due to the –C–H stretching from residual cysteine originating from the WS_2_ synthesis. A slight shift the C=O stretching vibration ~1650 cm^−1^ and ~1070 cm^−1^ the C-O-C of agar-agar matrix [20]. The peak at ~1400 cm^−1^ reflects the O–H deformation vibrations, and the peak at ~1300 cm^−1^ reflects the C–F stretching vibrations [19] of Ti_3_C_2_T_x_. The W-S stretching band near 600–700 cm^−1^ that confirms the presence od WS_2_ [21]. Taken together, these features indicate incorporation of the WS_2_/Ti_3_C_2_T_x_ hybrid filler into the hydrogel matrix and the formation of an effective interfacial contact without of degradation of the polymer network.

To study the phase composition and crystal structure, XRD analysis was performed (Figure 2b). The obtained diffraction data for the WS_2_/Ti_3_C_2_T_x_ composite were compared with the spectra of its individual components. For pure MXene, diffraction peaks were observed at 2θ ≈ 9.5°, 19.1°, 27.5°, 35.8° and 39.1°, corresponding to the (002), (004), (111), (200) and (110) planes of the Ti_3_C_2_T_x_ crystalline phase, indicating successful etching and exfoliation of the starting material. These data confirm the successful etching and exfoliation of MXene [22]. WS_2_ showed clear diffraction peaks at 2θ ≈ 14.2°, 28.3°, 32.8°, 44.4°, 58.4°, and 63.2°, indexed to the (002), (004), (001), (103), (008), and (112) planes of hexagonal WS_2_ (JCPDS Card No. 08-0237), indicating high crystallinity [23]. The WS_2_/Ti_3_C_2_T_x_ composite exhibited a superposition of the individual components’ diffraction peaks, without any additional peaks, indicating successful composite formation without structural degradation. Minor peak shifts and intensity variations (particularly in the 25–35° region) suggest interphase interactions or partial WS_2_ intercalation within the MXene layers. The X-ray diffraction results indicate the preservation of the crystalline structure of both components in the composite, which also points to the possible presence of synergistic interactions between the phases. The PAM-Ag-g/WS_2_/Ti_3_C_2_T_x_ hydrogel spectrum exhibits characteristic peaks of MXene and WS_2_, along with a broad amorphous peak at 2θ ≈ 23° from the PAM matrix [24], confirming the preserved crystallinity of the fillers and the polymer’s contribution.

Raman spectra (Figure 2c) showed characteristic vibrations for WS_2_. The out-of-plane mode A_1_g of sulfur at 458 cm^−1^ and the in-plane mode E_1_g associated with tungsten and sulfur atoms at 336 cm^−1^, which confirms the presence of the WS_2_ phase. The absence of distinct peaks for Ti_3_C_2_T_x_ phase could be attributed to the strong presence of Ti_3_C_2_T_x_ that reduces the overall crystallinity of the composite and leads to peak suppression in the Raman spectrum [25]. The presence of WS_2_/Ti_3_C_2_T_x2_ in the sample is complemented by XRD and FTIR results.

XPS was used to investigate the surface chemical composition and electronic states of the synthesized samples. The spectra (Figure 2d) demonstrate the presence of characteristic peaks of Ti, C, and O elements in the structure of pure Ti_3_C_2_T_x_. All spectra were calibrated using the C 1s peak at 284.8 eV. The spectrum of the WS_2_/Ti_3_C_2_T_x_ composite additionally contains peaks corresponding to W 4f and S 2p, which confirms the successful incorporation of WS_2_ into the Ti_3_C_2_T_x_ structure. The XPS data allowed us to clarify the elemental composition and types of chemical bonds in the WS_2_/Ti_3_C_2_T_x_ hybrid system. The Ti 2p spectrum contains peaks corresponding to Ti–O bonds at ~458.5 eV and ~464.3 eV, as well as S–Ti–C at ~455.1 eV, which indicate the existence of interphase interactions. The presence of the Ti–C peak confirms the preservation of the main MXene crystal structure. The C 1s spectrum contains signals corresponding to C–C, C–O/CH_x_, and C–Ti–T_x_ bonds, which are typical for functionalized Ti_3_C_2_T_x_, corresponding to ~284.8 eV, 286.2 eV, and 288.5 eV, respectively. The O 1s spectrum shows signals indicating the presence of chemical bonds C–Ti–O, O–Ti–O, and C–Ti–OH at ~529.9 eV, ~531.2 eV, and ~532.4 eV, correspondingly, which indicates the presence of –O and –OH functional groups on the surface of the material.

Analysis of the W 4f region reveals characteristic peaks corresponding to W–S bonds (~32.5 eV for W 4*f*_7/2_ and ~34.7 eV for W 4*f*_5/2_), as well as the W 5*p*_3/2_ peak at ~38.6 eV, which confirms the presence of WS_2_ in the composite. The S 2p spectrum contains peaks associated with S–W at ~162.3 eV (S 2p_3/2_) and ~163.5 eV (S 2p_1/2_) [26], as well as a weak peak at about ~165.6 eV, which is associated with the formation of Si–T or S–Ti bonds and indicates interfacial interactions [27]. These data confirm the formation of the WS_2_/Ti_3_C_2_T_x_ hybrid structure with the preservation of the crystallinity of the original components and the presence of interphase interactions.

**Figure 3:**
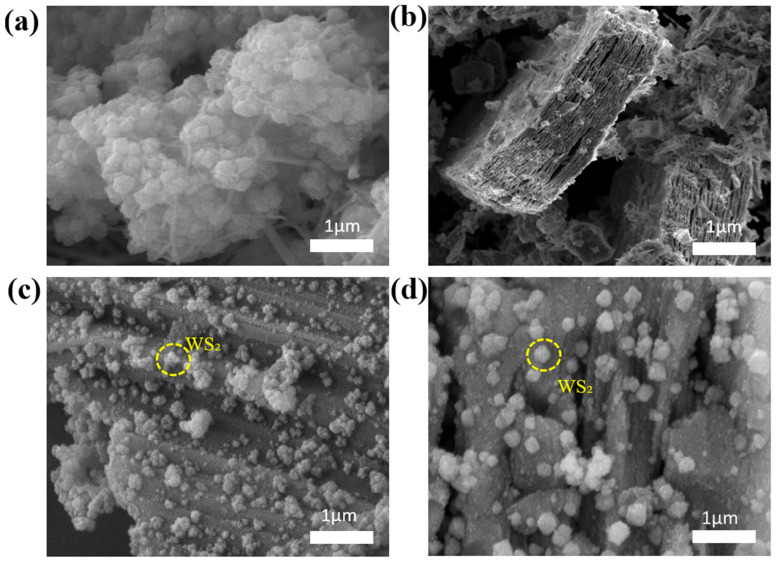
SEM images of: (**a**) WS_2_ nanoflowers, (**b**) Ti_3_C_2_T_x_, (**c**,**d**) WS_2_/Ti_3_C_2_T_x_ nanocomposite.

The morphology of the obtained Ti_3_C_2_T_x_, WS_2_, and WS_2_/Ti_3_C_2_T_x_ structures was analyzed by SEM, which confirmed the successful growth of both Ti_3_C_2_T_x_ and WS_2_ layered structures (Figure 3a–d). Figure 3a of pure WS_2_ shows a flower-like morphology consisting of numerous individual flower shapes. Ti_3_C_2_T_x_ shows an accordion-like morphology, as shown in Figure 3b, where the Ti_3_C_2_T_x_ layers can be seen separated from each other. The cross-section of the WS_2_/Ti_3_C_2_T_x_ heterostructure sample is shown in Figure 3c,d, which depicts the uniform distribution and inclusion of WS_2_ nanoflowers within the interlayer spaces of the MXene layers, leading to the formation of wrinkled WS_2_/Ti_3_C_2_T_x_ heterostructure nanosheets. 

**Figure 4:**
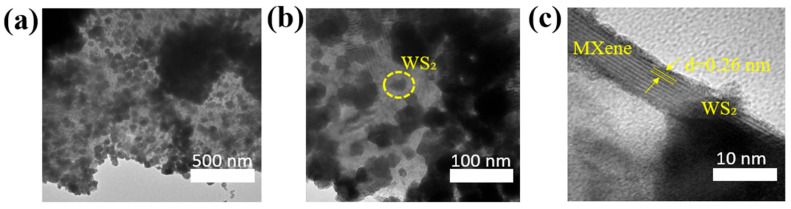
TEM image of WS_2_/Ti_3_C_2_T_x_ nanocomposite at different magnifications: (**a**) 500 nm, (**b**) 100 nm, (**c**) 10 nm.

The TEM images at different magnifications (Figure 4a–c) show that the flower-shaped WS_2_ nanostructures are uniformly distributed both on the surface and between the Ti_3_C_2_T_x_ layers. The measured interlayer distance in the heterostructure is 0.26 nm (Figure 4c), which corresponds to the Ti_3_C_2_T_x_ crystallographic plane [28].

### 3.2. Mechanical Properties of PAM-Ag-g/WS_2_/Ti_3_C_2_T_x_ Hydrogel

**Figure 5:**
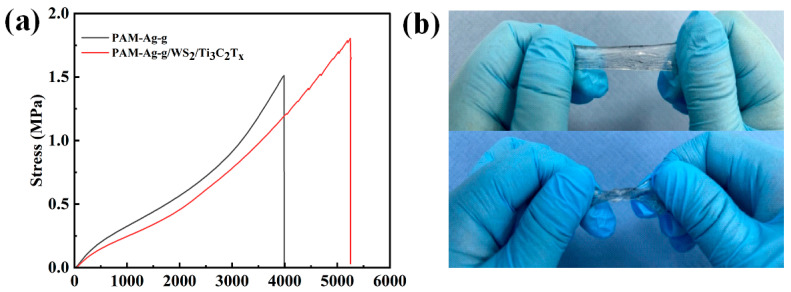
(**a**) Stress–strain curves of PAM-Ag-g и PAM-Ag-g/WS_2_/Ti_3_C_2_T_x_; (**b**) Visual demonstration of the strength of PAM-Ag-g/WS_2_/Ti_3_C_2_T_x_.

Reprehensive uniaxial tensile tests showed that the PAM-Ag-g/WS_2_/Ti_3_C_2_T_x_ hydrogel has improved mechanical properties compared to PAM-Ag-g (Figure 5a). This trend is consistent with hydrogen-bonding interactions formed upon adding Ti_3_C_2_T_x_ to the PAM-Ag-g matrix, with can enhance the hydrogels elasticity and ductility. Figure 5b shows that the composition retains high flexibility, strength, and resistance to destruction, which makes it promising for use in smart sensors, soft robotics, and biodegradable wearable devices.

**Figure 6:**
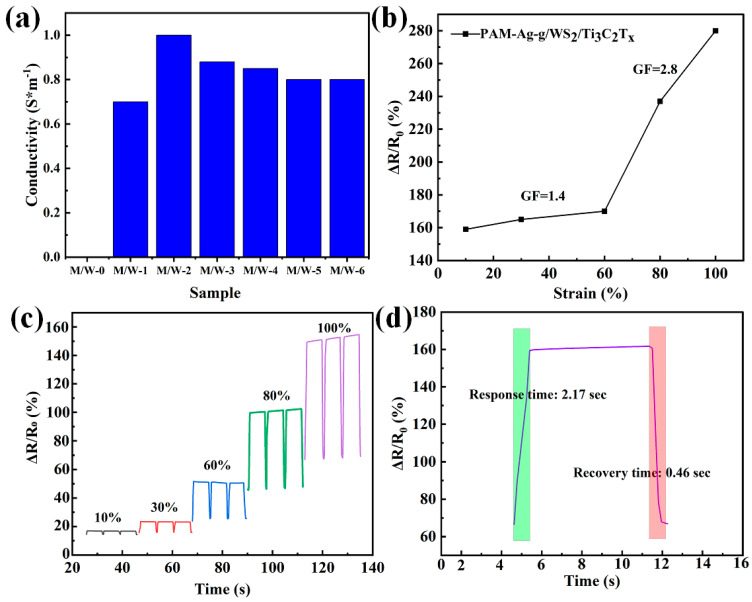
Electromechanical performances of the PAM-Ag-g/WS_2_/Ti_3_C_2_T_x_ hydrogel: (**a**) Conductivity of PAM-Ag-g/WS_2_/Ti_3_C_2_T_x_ with different MXene/WS contents; (**b**) Gauge factor of PAM-Ag-g/WS_2_/Ti_3_C_2_T_x_; (**c**) Variation in ΔR/R_0_ of the PAM-Ag-g/WS_2_/Ti_3_C_2_T_x_ at 10%, 30%, 60%, 80%, and 100%; (**d**) The response time and recovery time of the hydrogel sensor.

The addition of the WS_2_/Ti_3_C_2_T_x_ heterostructured nanocomposite to the PAM-Ag-g hydrogel made it possible to obtain an electrically conductive composite [29]. To determine the optimal conductivity, the WS_2_/Ti_3_C_2_T_x_ content was varied in the range of 0–1 wt.% (0, 0.2, 0.4, 0.6, 0.8, 1 wt.%). Based on the results of four-probe measurements, it was found that the maximum electrical conductivity (1.02 S m^−1^) was achieved at a content of 0.4 wt.MXene/WS_2_ (Figure 6a) exhibits enhanced performance with a further increase in concentration. Note that a similar decrease in conductivity was observed for other MXenes-based composites [30]. Consequently, the PAM-Ag-g/WS_2_/Ti_3_C_2_T_x_ hydrogel with 0.4 wt% WS_2_/Ti_3_C_2_T_x_ was selected for further investigation.

The strain Gauge factor (GF) of the strain gauge is calculated using the equation:

(1) $$GF=\frac{\left(\frac{∆R}{R_{0}}\right)}{\varepsilon }$$

where ΔR = R − R_0_, R_0_ is the initial resistance, and R is the resistance at a certain deformation, respectively, ε is the corresponding deformation [29].

The dependence of the relative change in electrical resistance (ΔR/R_0_) on the applied strain for the PAM-Ag-g/WS_2_/Ti_3_C_2_T_x_ composite demonstrates a linear character with calculated values of the sensitivity coefficient (Figure 6b) equal to 1.4 in the region of low strains and 2.8 at high degrees of stretching. The obtained indicators indicate the sensitivity of the material to mechanical effects, which confirms its potential as a strain gauge sensor. However, the magnitude of the Gauge factor is far beyond what has been reported for MXene-based sensors [30].

As shown in Figure 6c, an apparent change in resistance is observed during five extension-relaxation cycles carried out at different strain levels (10%, 30%, 60%, 80%, 100%). The relative resistance (ΔR/R_0_) increases gradually with increasing strain, with each loading stage being reproduced with high accuracy. This indicates that the PAM-Ag-g/WS_2_/Ti_3_C_2_T_x_ -based hydrogel effectively responds to mechanical loads over a wide range and demonstrates stable and reliable operation even under multiple strain cycles. The time plots of ΔR/R_0_ (%) change confirm the stability of the sensory response and its repeatability during long-term use.

When stretched to 100%, the sensor element showed a response time of 2.17 s and a rapid recovery of 0.46 s (Figure 6d), indicating the sensitivity of the material to external mechanical influences and its ability to quickly return to its original characteristics after the load is removed.

The working mechanism of the PAM-Ag-g/WS_2_/Ti_3_C_2_T_x_ strain sensor can be explained by the redistribution of charge carriers at the WS_2_/Ti_3_C_2_T_x_ heterointerface, driven by electron transfer from the WS_2_ layers to the highly conductive MXene surface, which is accompanied by the formation of a built-in electric field. This process is additionally enhanced by the interfacial dipole–dipole interactions between the –O/–OH groups of MXene and the external sulfur atoms of WS_2_, leading to some reduction in interface resistance, an increase in the initial conductivity, and a substantial enhancement of the sensor’s sensitivity to mechanical deformation.

### 3.3. Theoretical Simulations

**Figure 7:**
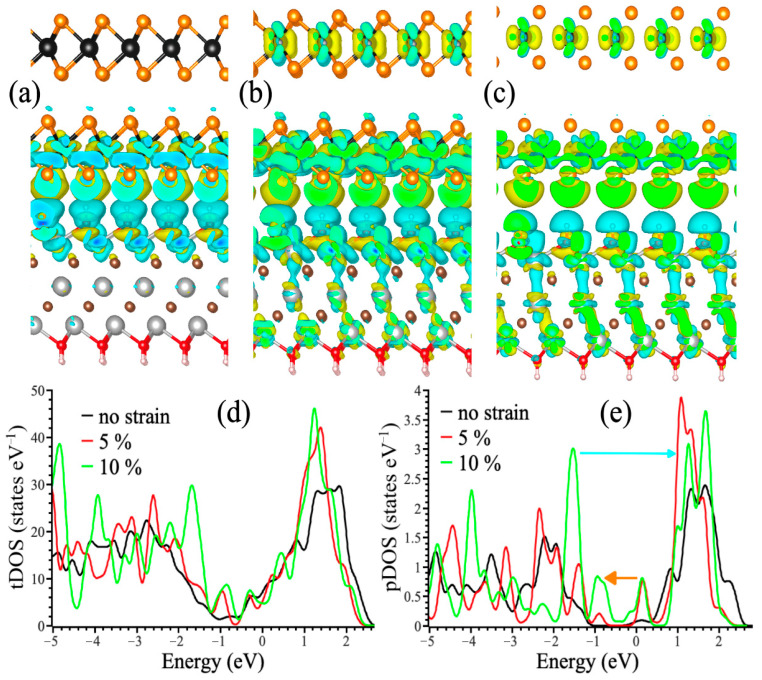
The changes in charge density distribution between the WS_2_ bilayer and the MXene substrate for no applied strain (**a**), and 5% (**b**) and (10%) of in-plane uniaxial stretching. The yellow and cyan “clouds” correspond with an increase and a decrease in eletron density, respectively. Tungsten atoms are shown in black, sulfur in light brown, oxygen in red, titanium in gray, carbon in dark brown, and hydrogen in pale pink. The isosurface level is the same on three panels (**a**–**c**). Total density of states of WS_2_/Ti_3_C_2_T_x_ interface (**d**) and W 5d partial (**e**) densities of states before and after applying in-plane stretching. Fermi energy set to zero. The blue and orange arrows in panel (**e**) indicate transitions corresponding to adsorption and emission of light, respectively.

To unveil the nature of the relatively modest values of the gauge factor and conductivity, we performed the simulation of the changes in the electronic structure upon formation of WS_2_/Ti_3_C_2_T_x_ composite and following substrate-induced stretching. For this purpose, we built a model interface between the bilayer of WS_2_ and the Ti_3_C_2_(OH)_2_ monolayer (Figure 7a). The formation of the interface led to the transfer of the electron density from the MXene substrate to WS_2_. Thus, a decrease in the conductivity can be associated with the formation of the interface between highly conductive MXene and a semiconductor.

Next, we checked how the substrate-induced strain affects the electronic structure of the composite. Figure 7b,c depict MXene to WS_2_ charge transfer for 5% and 10% in-plane uniaxial strain. As one can see, in-plane stretching leads to an increase in electron transfer from MXene to WS_2_. This massive redistribution of the charge density leads to the appearance of a pseudo-gap on the Fermi level (see the area with zero energy in Figure 7d). This explains the relatively modest values of the Gauge factor observed in the experiment. Note that visible changes are observed even in the second layer of WS_2_. This doping led to the appearance of distinct states inside the band gap (about −1 eV and +0.5 eV in Figure 7e). These and other states can be the source of distinct optical transitions, which are shown by arrows in Figure 7e. This arising of the particular states on the edges of conductive and valence bands can also be the source of catalytic activity in diselenides as it was shown in our recent work [31]. The combination of mechanical stability, relatively good conductivity, and strain-induced doping can be the source of manipulating the optical, catalytic, and photo-catalytic properties of semiconductive dichalcogenides attached to MXenes incorporated in polymer matrices.

## 4. Conclusions

In conclusion, the WS_2_/Ti_3_C_2_T_x_ hybrid structure was successfully synthesized. The combination of the conductive properties of Ti_3_C_2_T_x_ and the catalytically active surface of WS_2_ allowed the formation of a stable conductive network in the polymer matrix, which ensured the efficient transmission of an electrical signal under mechanical action. The composite hydrogel demonstrated high stretchability, improved mechanical properties and reliability under cyclic loads, and high signal reproducibility, indicating the stability of the sensor function of the hydrogel. The transfer of electrons explains the mechanism of operation of the strain gauge, confirmed by interface modeling, from the WS_2_ layers to the highly conductive Ti_3_C_2_T_x_ surface, accompanied by the formation of a built-in electric field. Thus, PAM-Ag-g/WS_2_/Ti_3_C_2_T_x_ hydrogel is a promising material for creating flexible strain gauges applicable in various applications such as motion and chemical sensing, optics and catalysis.

## Data Availability

The original contributions presented in this study are included in the article. Further inquiries can be directed to the corresponding author.

## References

[1] Pan J., Zhou X., Gong H., Lin Z., Xiang H., Liu X., Chen X., Li H., Liu T., Liu S. (2023). Covalently Functionalized MoS_2_ Initiated Gelation of Hydrogels for Flexible Strain Sensing. ACS Appl. Mater. Interfaces.

[2] Wang Q.H., Kalantar-Zadeh K., Kis A., Coleman J.N., Strano M.S. (2012). Electronics and Optoelectronics of Two-Dimensional Transition Metal Dichalcogenides. Nat. Nanotechnol..

[3] Randall J.N., Luscombe J.H., Bate R.T. (1994). Heterostructures and Quantum Devices.

[4] Feng S., Wang X., Wang M., Bai C., Cao S., Kong D. (2021). Crumpled MXene Electrodes for Ultrastretchable and High-Area-Capacitance Supercapacitors. Nano Lett..

[5] Wu J., Li Q., Shuck C.E., Maleski K., Alshareef H.N., Zhou J., Gogotsi Y., Huang L. (2022). An Aqueous 2.1 V Pseudocapacitor with MXene and V–MnO_2_ Electrodes. Nano Res..

[6] Zhu M., Du X., Liu S., Li J., Wang Z., Ono T. (2021). A Review of Strain Sensors Based on Two-Dimensional Molybdenum Disulfide. J. Mater. Chem. C.

[7] Wang J., Liu Y., Wang S., Liu X., Chen Y., Qi P., Liu X. (2021). Molybdenum Disulfide Enhanced Polyacrylamide-Acrylic Acid-Fe^3+^ Ionic Conductive Hydrogel with High Mechanical Properties and Anti-Fatigue Abilities as Strain Sensors. Colloids Surf. A.

[8] Zhang J., Su E., Li C., Xu S., Tang W., Cao L.N.Y., Li D., Wang Z.L. (2023). Enhancing Artifact Protection in Smart Transportation Monitoring Systems via a Porous Structural Triboelectric Nanogenerator. Electronics.

[9] Rasool F., Pirzada B.M., Talib S.H., Alkhidir T., Anjum D.H., Mohamed S., Qurashi A. (2024). In Situ Growth of Interfacially Nanoengineered 2D-2D WS2/Ti_3_C_2_Tx MXene for the Enhanced Performance of Hydrogen Evolution Reactions. ACS Appl. Mater. Interfaces.

[10] Han F., Chen S., Wang F., Liu M., Li J., Liu H., Yang Y., Zhang H., Liu D., He R. (2025). High-Conductivity, Self-Healing, and Adhesive Ionic Hydrogels for Health Monitoring and Human-Machine Interactions Under Extreme Cold Conditions. Adv. Sci..

[11] Lei H., Zhao J., Ma X., Li H., Fan D. (2021). Antibacterial Dual Network Hydrogels for Sensing and Human Health Monitoring. Adv. Healthcare Mater..

[12] Hou W., Sheng N., Zhang X., Luan Z., Qi P., Lin M., Tan Y., Xia Y., Li Y., Su K. (2019). Design of Injectable Agar/NaCl/Polyacrylamide Ionic Hydrogels for High Performance Strain Sensors. Carbohydr. Polym..

[13] Wang Y., Chen F., Liu Z., Tang Z., Yang Q., Zhao Y., Du S., Chen Q., Zhi C. (2019). A Highly Elastic and Reversibly Stretchable All-Polymer Supercapacitor. Angew. Chem. Int. Ed..

[14] Giannozzi P., Baroni S., Bonini N., Calandra M., Car R., Cavazzoni C., Ceresoli D., Chiarotti G.L., Cococcioni M., Dabo I. (2009). Quantum Espresso: A modular and open-source software project for quantum simulations of materials. J. Phys. Condens. Matter.

[15] Perdew J.P., Burke K., Ernzerhof M. (1996). Generalized gradient approximation made simple. Phys. Rev. Lett..

[16] Barone V., Casarin M., Forrer D., Pavone M., Sambi M., Vittadini A. (2009). Role and effective treatment of dispersive forces in materials: Polyethylene and graphite crystals as test cases. J. Comput. Chem..

[17] Vanderbilt D. (1990). Soft self-consistent pseudopotentials in a generalized eigenvalue formalism. Phys. Rev. B.

[18] Zhao L., Zheng Y., Wang K., Lv C., Wei W., Wang L., Han W. (2020). Highly Stable Cross-Linked Cationic Polyacrylamide/Ti_3_C_2_Tx MXene Nanocomposites for Flexible Ammonia-Recognition Devices. Adv. Mater. Technol..

[19] Chen X., Zhao Y., Li L., Wang Y., Wang J., Xiong J., Yu J. (2020). MXene/Polymer Nanocomposites: Preparation, Properties, and Applications. Polym. Rev..

[20] Wahba M.I., Hassan M.E. (2017). Agar-Carrageenan Hydrogel Blend as a Carrier for the Covalent Immobilization of β-D-Galactosidase. Macromol. Res..

[21] Vattikuti V.S.V., Chan B. (2015). Effect of CTAB Surfactant on Textural, Structural, and Photocatalytic Properties of Mesoporous WS_2_. Sci. Adv. Mater..

[22] Yang J., Voiry D., Ahn S.J., Kang D., Kim A.Y., Chhowalla M., Shin H.S. (2013). Two-dimensional hybrid nanosheets of tungsten disulfide and reduced graphene oxide as catalysts for enhanced hydrogen evolution. Angew. Chem. Int. Ed..

[23] Chen R., Cheng Y., Wang P., Wang Y., Wang Q., Yang Z., Tang C., Xiang S., Luo S., Huang S. (2021). Facile synthesis of a sandwiched Ti_3_C_2_T_x_ MXene/nZVI/fungal hypha nanofiber hybrid membrane for enhanced removal of Be(II) from Be(NH_2_)_2_ complexing solutions. Chem. Eng. J..

[24] Miah M.Y., Halder S., Saikat S.P., Dewanjee S., Ashaduzzaman M., Bhowmik S. (2025). Microwave-assisted one-step synthesis of polyacrylamide/NiO nanocomposite for biomedical applications. RSC Adv..

[25] Wang Q., Liu A., Qiao S., Zhang Q., Huang C., Lei D., Shi X., He G., Zhang F. (2023). Mott-Schottky MXene@WS_2_ Heterostructure: Structural and Thermodynamic Insights and Application in Ultra Stable Lithium−Sulfur Batteries. ChemSusChem.

[26] Zhang B., Luo C., Deng Y., Huang Z., Zhou G., Lv W., He Y.B., Wan Y., Kang F., Yang Q.H. (2020). Optimized Catalytic WS_2_–WO_3_ Heterostructure Design for Accelerated Polysulfide Conversion in Lithium–Sulfur Batteries. Adv. Energy Mater..

[27] Yuan Z., Wang L., Li D., Cao J., Han W. (2021). Carbon-Reinforced Nb_2_CTx MXene/MoS_2_ Nanosheets as a Superior Rate and High-Capacity Anode for Sodium-Ion Batteries. ACS Nano.

[28] Wang X., Luo D., Wang J., Sun Z., Cui G., Chen Y., Wang T., Zheng L., Zhao Y., Shui L. (2021). Recent Advances in Layered Transition Metal Dichalcogenides for Hydrogen Evolution Reaction. Angew. Chem. Int. Ed..

[29] Biswas M.C., Chakraborty S., Bhattacharjee A., Mohammed Z. (2021). 4D Printing of Shape Memory Materials for Textiles: Mechanism, Mathematical Modeling, and Challenges. Adv. Funct. Mater..

[30] Leong W.X.R., Al-Dhahebi A.M., Ahmad M.R., Saheed M.S.M. (2022). Ti_3_C_2_Tx MXene-Polymeric Strain Sensor with Huge Gauge Factor for Body Movement Detection. Micromachines.

[31] Boukhvalov D.W., Chuchvaga N.A., Rakhimzhanov M.K., Shongalova A., Serikkanov A.S., Osipov V.Y. (2025). The Effect of the Metal Impurities on the Stability, Chemical, and Sensing Properties of MoSe_2_. Surfaces.

